# Food Attention Bias: appetite comes with eating

**DOI:** 10.1186/s40337-021-00489-3

**Published:** 2021-10-19

**Authors:** Lilac Lev-Ari, Hamutal Kreiner, Omer Avni

**Affiliations:** 1 Behavior Sciences, Ruppin Academic Center, Emek Hefer, Israel; 2 The Lior Tsfaty Center for Suicide and Mental Pain Studies, Ruppin Academic Center, Emek Hefer, Israel; 3 Gerontological Clinical Psychology, Ruppin Academic Center, Emek Hefer, Israel

**Keywords:** Food Attention Bias, Cognitive reappraisal, Body dissatisfaction, Disordered eating, Moderation

## Abstract

**Objective:**

Cognitive interventions may be effective in weight loss. The purpose of this study was to assess if cognitive reappraisal (CR; reframing the meaning of a stimulus in order to change the resulting emotional response), can reduce food attentional bias (FAB) using the Visual Dot Probe (VDP) paradigm.

**Method:**

126 participants (age 27 ± 5.8) were randomly assigned either to a CR or to a control (CN) group. After assessing baseline VDP levels for FAB, participants either wrote sentences that discourage eating fattening food or neutral sentences. Next, all participants performed the VDP post intervention. Participants also self-reported on disordered eating behaviors and their height and weight were charted. We hypothesized that CR would reduce FAB and that disordered eating would moderate the association between group and FAB.

**Results:**

FAB decreased post intervention, specifically in the CR group. The bulimia sub-scale showed an interaction between bulimic eating, time and group. Among those who were high on the bulimia scale, the CR group showed lower FAB post-intervention compared to the CN group.

**Discussion:**

This study suggests that CR may decrease the attentional bias toward high-calorie food compared to other strategies in the general population and among people with high bulimia measures, in particular.

**Plain English summary:**

Obesity has a negative impact on many aspects of life and much research is dedicated to trying to better understand behaviors concerning obesity. People are prone to focus their attention on things that are of importance to them, such as food. When people focus their attention on food, we call this Food Attention Bias (FAB). Cognitive reappraisal (CR) interventions involve the person's conscious cognitive change of the meaning of the situation aiming to consequently change the emotional response to it, such as saying to yourself “I shouldn’t eat this because I don’t want to get fat”. CR has been found to be helpful in lowering FAB using brain imagining techniques but has not yet been studied in cognitive processes. Our study used a Visual dot probe paradigm (VDP) to assess the efficacy of CR on lowering FAB. Two groups, one using CR and a control group were assessed twice on FAB, using the VDP paradigm. Compared to the normal condition, the CR intervention helped reduce FAB. This reduction was especially significant for people with a higher tendency for bulimic behavior. The VDP paradigm, utilizing CR. can be expanded to help build an intervention aimed at reducing FAB over time. This, in turn might bring to weight reduction. People with bulimic tendencies might especially benefit from CR interventions when dealing with weight loss.

## Introduction

The shortage of time, and the endless selection of food products can lead people to eat regardless of their hunger and energy needs and consume fast food that has lage caloric value [6, 33, 34]. Research shows that the appearance of appetizing food products attracts attention and may be enough to trigger a person's food intake for pleasure only, regardless of existential needs [8, 34], which can lead to obesity.

### Obesity and disordered eating

While the global average of obese people stands at 10.7% of men and 15.2% of women over the age of 18, in Israel the picture is even more bleak. Over 23% of men and 27% of women in Israel (age 18 and up) are considered overweight [41]. Obesity increases the risk of diseases such as diabetes, various types of cancer and heart disease and is now considered a global epidemic [25, 33]. This study examines a possible way of coping with Food Attention Bias (FAB) that, together with other factors, may lead to obesity. The study examines the impact of a cognitive strategy based on a stimulus re-evaluation technique, on people's susceptibility to FAB to tempting food stimuli, aiming to promote the development of a tool for coping with obesity.

Obesity is defined as a condition of abnormal or excessive fat accumulation in adipose tissue, to the extent that health is impaired [30]. The most widely used tool for measuring and diagnosing obesity is the body mass index (BMI) due to its simplicity and low cost [24]. A value of 30 or higher is considered obese, 25–29.9 is overweight and values that are in between 18.5 and 24.9 indicate normal weight [24, 30].

Many factors contribute to the ever-growing epidemic of obesity. High-calorie food is accessible because of its low cost and immediate availability [42]. Genetic [31], and social factors [42] also contribute to the risk of obesity.

Obesity may be related to disordered eating. Disordered eating refers to a wide spectrum of unhealthy eating behaviors all of which are not dangerous enough to warrant a psychiatric diagnosis [36]. Some of these behaviors are diet-related, such as an unsupervised diet that includes strict calorie intake, while other behaviors include unhealthy eating, such as consuming a large amount of high-caloric food while skipping meals. In addition, anorectic or bulimic behaviors such as taking laxatives or diet pills, vomiting, and periodicity of binge eating and dieting are also considered to be disordered eating behaviors [18].

Brain imaging studies have shown an association between the Mesocorticolimbic pathways, associated with providing rewards and various cognitive processes, and overeating [1, 14]. This pathway has been previously linked to the term 'appetitive motivation', which means increasing behavioral orientation toward goals that have pleasant and positive hedonistic effects such as eating, drinking alcohol or having sex. The hedonistic effects can be related to one's subjective experience and emotional impact of action [5].

Previous studies have found that overweight individuals show greater brain activity in more extensive reward areas than thin individuals, and reduced brain activity in inhibitory areas in response to food images, especially high-calorie food [43]. Accordingly, it has been suggested that the tension between appetitive motivation and cognitive inhibition leads to a person's eating behavior, so that lack of control and goal-directed behavior of food consumption, especially calorie-rich food, which is of greater hedonistic value, will lead to weight gain, overweight and obesity [2, 26, 27].

### Cognitive strategies for intervention in eating behaviors

Due to the many negative effects of obesity noted above, and the expansion of the phenomenon to the point of being defined as an epidemic in recent years, the need to develop weight loss treatment methods and maintain proper body weight has increased [38]. One of these ways, which addresses the uncontrollable desire to eat foods of great calorific value, is a cognitive reappraisal (CR) exercise. CR interventions involve the person's conscious cognitive change of the meaning of the situation aiming to consequently change the emotional response to it [11]. Hence, it may strengthen one's cognitive control when encountering high-calorie food, thereby weakening the appetitive motivation. This strategy has previously been supported by findings demonstrating that cognitive interventions contribute to a decrease in food cravings through active control of one's way of thinking about food [11, 40].

CR aims to change how one thinks about emotionally stimulating cues, such as high-calorie food cues. In most studies using brain imaging, the instructions for practicing this strategy are not detailed, however, [11] describes the following procedures: (1) "Imagine that currently you are very replete, (2) focus on the negative consequences of eating this food (stomach ache or weight gain for example), (3) Remind yourself that you can keep this dish for later, (4) Imagine something bad happened to the dish (let's say someone sneezed on it)." Each participant had to choose one of this proposed CR strategies or create an alternative strategy that would be applicable in the real world and use it throughout the experiment. Subsequently, the researchers found that there was a decrease in the desire to eat high-calorie food, with no significant difference between the strategies proposed by the research team and those created by the participants themselves [11].

Functional Magnetic Resonance Imaging (fMRI) studies of smokers and studies of people with normal BMI found that using CR when given the long-term negative effects of eating high-caloric food has reduced the desire for high- caloric food [16, 34]. In addition, using CR has been shown to increase activity in inhibitory areas (such as the Gyrus and Ventrolateral Prefrontal Cortex) in exposure to high-fat or sugar-rich food, and attenuate activity in attentional areas (such as the Precuneus and Posterior Cingulate Cortex). These findings suggest that CR can suppress appetitive motivation and reduce unhealthy food intake in overweight individuals [35, 43].

### Changes in attentional bias as a measure of the impact of cognitive strategies

Attentional Bias (AB) is a state of automatic and excessive attention to specific stimulation [19]. Attention bias towards food is a specific case of AB, called Food Attentional Bias. Berridge [3] proposed the model of food reward, which holds that unhealthy eating is a behavioral response to such FAB. According to this model, unhealthy food cues capture more attention as they are perceived as more attractive, rewarding, and tasteful [32]. FAB has been linked to people's inability to resist the temptation of food [12] suggesting that obese people will have greater FAB. FAB leads to faster processing of food-related information in obese individuals relative to non-obese individuals [13].

In this study, we used the Visual Dot Probe (VDP) to assess FAB. This procedure is commonly used to measure AB toward various stimuli, such as smoking and alcohol [9, 37], See Methods section). A study that used the VDP procedure as an indicator of FAB, found that all participants exhibited FAB, but obese individuals showed an increased FAB compared to participants without obesity [28, 29]. This suggests that the VDP task provides a sensitive measure of FAB [13].

### This study

The main goal of the current study was to examine in what ways FAB is modulated by cognitive-behavioral procedures used for regulating food consumption. To this end we tested participants' FAB before and after they performed a CR or a control procedure and analyzed their effects on FAB. Participants were divided into two groups: In the CR group, they performed a cognitive reappraisal procedure and in the control group they performed a neutral task (CN). All groups performed a computerized VDP task before and after the intervention. In this task, two stimuli were briefly presented on the screen and participants only watched them. These stimuli included the target stimulus—either a word or a picture of *food*, and a neutral stimulus, either a word or a picture of an *animal*. Immediately after the word or picture disappeared, a dot appeared on the screen where either the target or the neutral stimulus had been presented. Participants were asked to press a key to indicate the location of the dot, and their reaction time (RT) was recorded. The difference between RT on incompatible (the dot appeared in the same position as the food stimulus) and compatible (the dot did not appear in the same position as the food stimulus) trials served as the FAB score.

Following Giuliani et al.’s [11] study, in the intervention procedure, participants were required to write and memorize five sentences, while they were watching a set of pictures that included high-calorie appetizing food products. Participants in the CR group were instructed to write sentences about the negative consequences of eating high-calorie foods whereas participants in the CN group were required to write and memorize five neutral sentences about their day. Following the intervention procedure, all participants performed the VDP task again, with a different set of stimuli. In the last phase of the study the participants self-reported on demographics and eating behaviors.

We hypothesized that:

1. In the CR group, post intervention FAB scores will be lower than pre-intervention scores. In addition, following the intervention, the CR intervention group would show reduced FAB compared to the control group.
2. The CR intervention will have a greater impact on FAB levels among participants with higher disordered eating behaviors than participants with lower disordered eating behaviors.

## Method

### Participants

The study included 126 non-vegetarian non-vegan participants recruited from the community or from undergraduate students in an Israeli college earning course credit. The sample consisted of 35 men and 91 women. Participants were randomly assigned to the one of the intervention groups. The CR intervention group included 18 male and 49 women, with mean age 26.49 ± 5.57 and mean BMI 24.25 ± 4.78, and the CN group included 17 male and 42 women, with mean age 27.56 ± 6.14 and mean BMI 24.45 ± 4.76Thirty-six (28.6%) of the participants were underweight, 74 (58.7%) had normal weight and 16 (12.7%) were overweight. They were evenly distributed between the groups. There were no statistical differences between the groups in any of these variables and all variables were normally distributed. Table 1 shows all indices by group.

**Table 1:** Demographic and self-reported indices by group

	CR Mean (SD) N = 67	CN Mean (SD) N = 59	ALL Mean (SD) N = 126	Statistical significance
BMI	24.25 (4.78)	24.45 (4.76)	24.24 (4.5)	t_(df)_ = .13, NS
Age	26.49 (5.57)	27.56 (6.14)	27.23 (5.93)	t_(df)_ = .78, NS
Diet	2.86 (.89)	2.73 (.90)	2.79 (.89)	F_(1,122)_ = .65, NS
Bulimia	2.37 (.73)	2.26 (.68)	2.31 (.70)	F_(1,122)_ = .74, NS
Oral Control	1.83 (.57)	1.89 (.62)	1.86 (.59)	F_(1,122)_ = .32, NS
EAT-26 Total	2.35 (.55)	2.29 (.53)	2.35 (.53)	F_(1,122)_ = .37, NS

Diet = Diet subscale EAT-26; Bulimia = Bulimia subscale EAT-26; Oral Control = Oral Control subscale EAT-26. The differences between groups for age and BMI were assessed using two independent t-tests and the difference between groups for EAT-26 indices were assessed using a 2*4 MANOVA test (with group as the independent variable and EAT-26 indices as the dependent variables)

### Design and procedure

The study was constructed as a 2×2 mixed-design experiment, manipulating the intervention (cognitive reappraisal (CR) or control (CN)) as a between participant variable and the FAB testing time (pre- or post-intervention) as a within-participant variable. BMI and disordered-eating were used as additional possible covariates.

Participants were invited to take part in a study a perception and attention study related to food. In view of previous findings indicating that FAB may be increased by hunger (e.g., by fasting [35, 43], participants were asked to eat about two hours before they took part in the study, but not during the last hour before they started their participation, and their compliance was verified. The study was individually conducted in a quiet room, and the session lasted about 40 min. Participants performed the tasks in the following order: (1) Pre-test VDP task (2) Intervention procedure (3) Post-test VDP task (4) Computerized questionnaire including Disordered Eating, demographic variables and self-reported BMI. At the end of the experiment, participants were provided with information about the study and the e-mail addresses of the authors for future inquiries.

### Tools

#### Food Attention Bias score (FAB): the VDP task

**Fig. 1:**
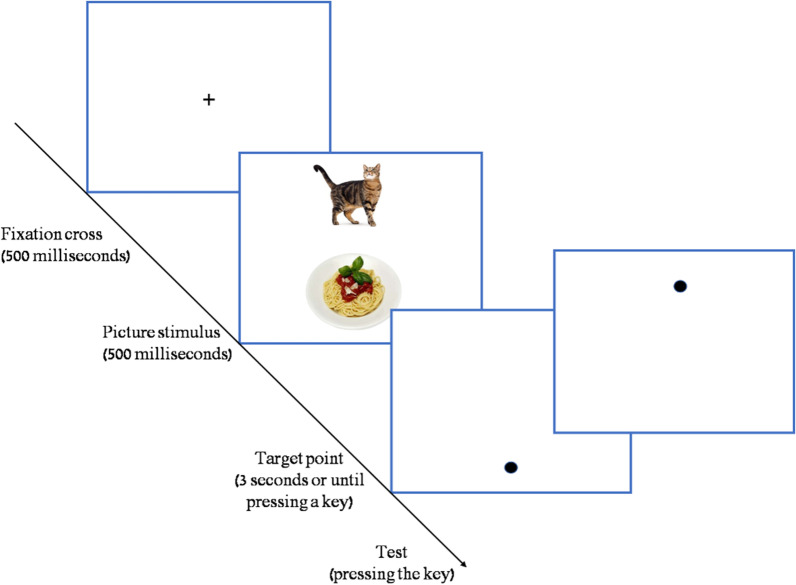
Three stages of the VDP picture task: fixation cross, animal-food picture, compatible / incompatible dot position

To test FAB, a VDP task (following [13] and [15] comprised of two blocks was conducted one of them use pictures and the other words. In the picture block, each step in the VDP task included three phases (see Fig. 1). In the first phase, a fixation cross appeared in the center of the screen for 500 ms (ms.), and participants were asked to focus on it. Once they were focused, the pressed a key to move to next phase. On the second phase, two pictures appeared on the screen for 500 ms., such that on half the trials an animal picture was displayed above the center of the screen and a picture food below, on the other half the food picture was displayed above the center of the screen and the animal below. Participants were asked to watch the pictures, but they were not required to respond in any way. In the third phase, a dot was displayed on the screen either on the same spatial location where the food picture was previously displayed—in the *compatible* condition, or in the location where the animal picture appeared—in the *incompatible* condition. The order of compatible and incompatible trials was random. Participants were asked to respond as fast as possible to indicate the location of the dot by pressing the V key on the keyboard when the dot was at the bottom of the screen or the U key when the dot was at the top of the screen, and their reaction-time (RT) was recorded. The interval between steps was 1000 ms. In the word VDP block the same procedure was used, but instead of pictures, words of food and animals were presented. We decided to use both images and words because the literature was inconclusive as to which stimuli are more effective in eliciting FAB [15]. The order of the pictures and words blocks was randomized and counter-balanced between participants. Each participant took ten training steps before starting the pre-test phase to make sure the instructions were understood. Following the training, the pre-intervention test began, with five additional training steps followed by eighty test steps. The post-intervention FAB test that began following the intervention procedure was similar in all aspects but used different pictures and words. The FAB score was calculated from the participants' RT as the difference between RT on *incompatible* steps and RT on *compatible* steps.

#### Pictures

Forty pictures of foods and 40 pictures of animals were used for the picture FAB test (for a full list, please contact authors). Pictures of high-calorie foods such as croissants and hamburgers were selected from the materials of Blechert et al. [4]. Pictures of favorable animals, typically not eaten in western cultures (e.g., parrot, hippo) were selected from Kemps et al.'s [15] study. The animals were selected because, like food items, this category is generally considered attractive [15]. To verify that the pictures were valid and suitable for Israeli participants we conducted a pilot study in which participants (N = 100 were asked to rate the pictures of the food and animals on three 1–7 scales, indicating (a how clear was the picture, (b to what extent it attracted attention; and (c how appealing it is for eating. All food pictures were rated above 4 on the "appealing for eating" scale (except for one item with less than 4 but still higher than 3. By contrast, all animal pictures were rated below 3 on that scale, confirming that they were not associated with food.

#### Words

Forty words referring to high-calorie foods, such as fries and chocolate [4, 34], and 40 words referring to favorable animals typically not eaten in western cultures (e.g., iguana) served for the word FAB-test. As the test was conducted in Hebrew, we could not use the English stimuli used in previous studies. Hence, we conducted a pilot to evaluate how appealing to eat they were. Participants (N = 100 who did not participate in the main experiment) were presented with a list of 60 words of food and 60 words of animals and asked to rate them on a 1–7 scale indicating to what extent they were appealing for eating. Finally, the food pictures rated as highest on the appealing to eat scale, and the animal pictures rated as lowest were selected for the VDP task. The words were presented in black on a white background. They were displayed above each other horizontally centered at an equal distance (40 mm) from the center of the screen.

The 40 pictures and 40 words selected for the study were divided into two lists such that half the participants were presented with one list in the pre-test and the other one in the post-test, and the order was reversed for the other participants (For the full list, please contact authors. “Appendix” details means and SDs, along with the t-tests values for the differences between the lists in terms of the pictures and words ratings (i.e., picture clarity, attention attraction appeal for eating and word frequency). The results show some differences between the lists, but as these differences were revealed only for the animals' stimuli, we did not recalibrate the lists.

### Cognitive intervention procedures

#### Cognitive reappraisal

Participants were instructed to write down five sentences about negative consequences of eating high-calorie foods related to health risk, body image etc. The experimenter gave them examples such as “This food is not healthy for me” or “I will regret eating this later on”. Subsequently, they were presented with 5 pictures of high-calorie foods on the computer monitor and were asked to rehearse aloud the sentences while watching the pictures. They were then asked to rewrite the sentences repeatedly for 5 min. Then another set of high-calorie foods was displayed, and they had to rewrite the sentences again, and this cycle repeated 4 times for a duration of 20 min.

#### Control

In the control group participants were instructed to write neutral sentences relating to their day-to-day life, which were not emotionally stimulating. The experimenter gave them examples such as “Today I took the bus to school” or “I brushed my teeth when I got up this morning”. They were then asked to use the sentences suggested to them or make up their own and rewrite them repeatedly for 5 min.

### Disordered eating

Disordered eating was measured using the Eating Attitudes Test Questionnaire (EAT-26; [10]. The EAT-26 contains three sub scales: Diet (13 items such as: "I feel very guilty after eating"), Bulimia (7 items such as: "I had binge-eating episodes I feared I would not be able to stop") and Oral control (6 items such as: "I feel others would prefer I eat more"). Each item is rated on a 6-point Likert-type scale ranging from 1 (never) to 6 (always). We focus on the bulimia subscale because it reflects over eating, and may be expected to be more closely associated to FAB. The Hebrew version of EAT-26 has been found successful in distinguishing between people with or without eating disorders. Internal reliability for the original and Hebrew versions (Cronbach's alpha = 0.81 for the entire questionnaire; [17, 44] is comparable to previous findings [10] Cronbach's alpha = 0.83 for the entire questionnaire,Test–Retest reliability Cronbach's alpha = 0.80).

### BMI

Participant’s height and weight where charted. BMI was calculated by dividing weight in Kilograms by height in meters squared.

### Data analysis

All indices were assessed for normal distribution attributes, and all fell within accepted parameters. T-tests were used to test the significance of the differences between the groups. PPearson correlations were used to assess associations between quantitative variables. To test the hypothesis that the intervention influenced FAB, we conducted a two-way mixed ANOVA on the FAB scores with time as a within-participant variable and group as a between-participant variable. A three-way ANOVA tested the significance of the effects of time, intervention, Disordered Eating and the interactions between them.

## Results

Preliminary data assessing the EAT-26 total score and subscales across the different groups is reported in Table 2. For the CR intervention group the mean score on EAT-26 was 2.35 ± 0.55, and for the CN group 2.29 ± 0.53. Fourteen (11.1%) of the participants met the EAT-26 cutoff score for eating disorders. They were evenly distributed across groups.

**Table 2:** EAT-26 indices by group

	CR Mean (SD) N = 67	CN Mean (SD) N = 59	ALL Mean (SD) N = 126	Statistical significance
Diet	2.86 (.89)	2.73 (.90)	2.79 (.89)	F_(1,122)_ = .65, NS
Bulimia	2.37 (.73)	2.26 (.68)	2.31 (.70)	F_(1,122)_ = .74, NS
Oral Control	1.83 (.57)	1.89 (.62)	1.86 (.59)	F_(1,122)_ = .32, NS
EAT-26 Total	2.35 (.55)	2.29 (.53)	2.35 (.53)	F_(1,122)_ = .37, NS

Diet = Diet subscale EAT-26; Bulimia = Bulimia subscale EAT-26; Oral Control = Oral Control subscale EAT-26. The differences between groups for EAT-26 indices were assessed using a 2*4 MANOVA test (with group as the independent variable and EAT-26 indices as the dependent variables)

Data analysis was based only on the correct responses, incorrect responses (less than 5% per condition) were not included in the analysis. For each participant, the FAB score was calculated by subtracting the mean response times in the compatible condition (the dot position was incompatible with the food position) from the mean response times in the incompatible condition (the dot position was compatible with the food position), for the pre-test and for the post-test separately. As we did not hypothesize a difference between words and pictures, and there was no significant difference between them in the FAB scores we averaged the scores across all stimuli to generate a united FAB score. The average reaction times (in Milliseconds) and standard deviations of the different experimental conditions are shown in Table 3. Note, that positive FAB values indicate that more attention was drawn to the food items and less to the neutral ones, values around zero indicate no bias, and negative FAB values indicate attentional avoidance from food items (e.g., [39].

**Table 3:** Average FAB scores (in milliseconds) pre- and post-intervention by intervention group

	CR Mean (SD) N = 67	CN Mean (SD) N = 59	ALL Mean (SD) N = 126	t_(df)_
Pre-test	1.55 (20.99)	− 3.37 (20.87)	−.75 (21.00)	t_(124)_ = − 1.32, NS
Post-test	− 5.66 (20.93)	− .50 (17.28)	− 3.25 (19.41)	t_(124)_ = 1.50, NS

BMI ranged between 16.53–38.29 with a mean of 24.4 and SD = 4.75. BMI was positively correlated with the bulimia subscale of the EAT-26 (r = 0.33, *p* < 0.05). BMI and FAB scores were not correlated (r = − 0.14, *p* = 0.13). No other correlations were found pre-intervention.

### Effect of intervention procedure on FAB

Hypothesis 1: *The CR group would show a reduction in FAB at the end of the intervention, compared to the CN group.*

To examine that there were no differences between the groups before the intervention, a T-Test for independent samples was conducted on the pre-test FAB scores. Although the scores presented in Table 3 show a small difference, the T-Test shows that this difference was not significant. To test the hypothesis that the intervention (CR vs. CN) influenced FAB, we conducted a two-way mixed ANOVA on the FAB scores with time as a within-participant variable (pre/post intervention) and group as a between-participant variable (CR / CN). No main effect for time was found, and no main effect was found for group. Critically, the interaction between group and time was significant (F_(2,124)_ = 5.13, *p* < 0.05), as hypothesized. Post Hoc tests revealed a decrease in FAB in the CR group post-intervention compared to pre-intervention (difference = 7.21, *p* < 0.05), in accordance with our hypothesis. However, no significant difference was found between pre- and post-interventions (difference = 2.87) in the CN group (see Table 3), as expected.

### Disordered eating

Hypothesis 2: *The CR intervention will have a greater impact on FAB levels among participants with higher disordered eating behaviors than participants with lower disordered eating behaviors, revealing an interaction between the intervention group (CR and CN) and disordered eating in their influence on the reduction in FAB following the intervention.*

**Fig. 2:**
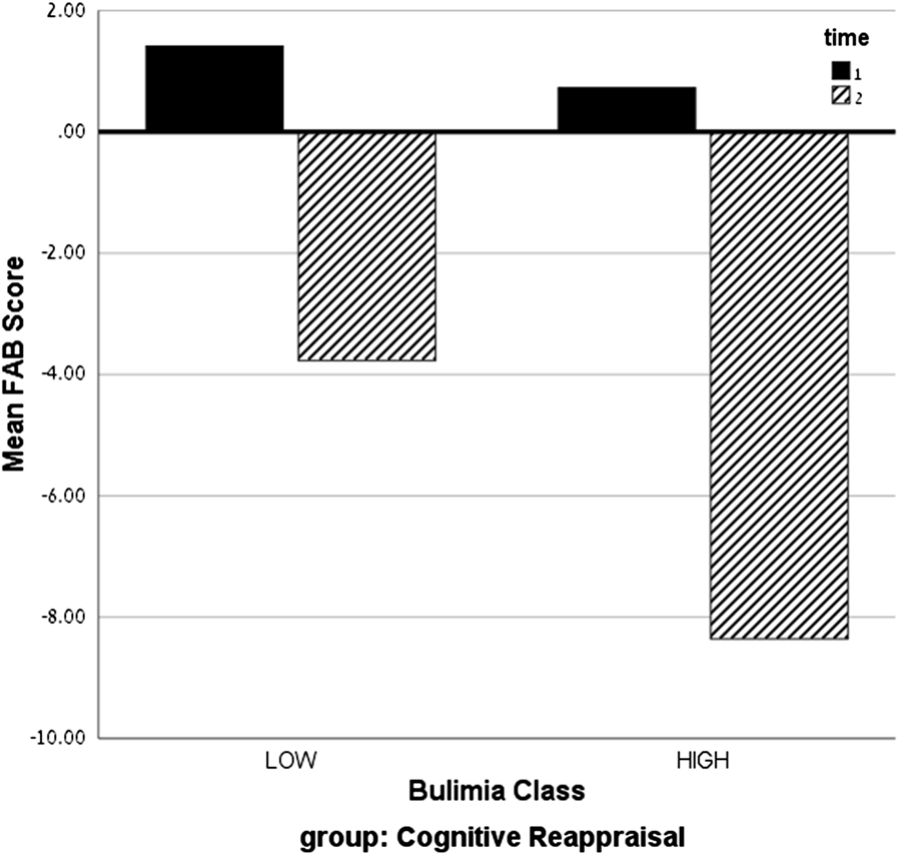
Mean FAB scores in the Cognitive Reappraisal group by time (pre/post intervention) and bulimia class (low/high). Note: time 1 = pre-intervention; time 2 = post-intervention

To test this hypothesis, we classified participants to high or low on the Disordered Eating scale using a median split. A three-way ANOVA (time × intervention × Disordered Eating) revealed no significant effect for the Disordered Eating total score or for the Diet and Oral restraint subscales, or for their interactions with time or intervention. Hence, we focused on the bulimia subscale of the EAT-26, which showed a difference between intervention groups. We classified participants to high or low on the bulimia subscale using a median split and conducted a three-way ANOVA (time × intervention × bulimia). The analysis revealed a significant three-way interaction (F_(1,118)_ = 5.66, *p* < 0.05, see Fig. 2). And a two-way interaction between time and group (F_(1,118)_ = 5.36, *p* < 0.05). To further understand this interaction regarding our hypothesis we conducted a two-way ANOVA for the CR group. The results show a significant interaction between time and bulimia class (F_(1,63)_ = 5.92, *p* < 0.05) with a larger intervention effect for the high bulimia class (difference = 9.01) compared to the low bulimia class (difference = 5.18), as can be seen in Fig. 2.

## Discussion

The purpose of the present study was to examine the effects of a cognitive regulation strategy on attentional bias toward food cues (FAB), and the susceptibility of participants with different levels of disordered eating scores to such intervention. Previous studies in the field typically examined separately the relationship between cognitive strategies and changes in attention bias toward food [11, 15, 40], or between cognitive strategies and their relation to disordered eating [7, 22, 23]. The novelty of the present study is that it combines, in one experimental set, a study of the effect of CR on FAB and their relationship to disordered eating. Moreover, while previous studies used either self-report measures or neurophysiological brain responses, we use the VDP task which is an objective, highly sensitive behavioral measure of FAB.

Our key hypothesis was that CR will reduce participants' FAB levels. This hypothesis was confirmed, and thus our study extends previous findings that were obtained in different methods (i.e. fMRI; [34, 43]. While past studies have primarily focused on changes in cerebral activity which are not always easy to associate with particular behavior, the results of the current study indicate that CR strategy has a direct effect on human behavior. The CR strategy reduced FAB as reflected in the VDP task compared to the control group suggesting that such interventions can reduce attentional focus on fattening food. Furthermore, we found that there were no differences between both groups on self-reported BMI or EAT-26 subscales and total score, assuring that these were not cofounding factors. Critically, the negative FAB scores post-intervention indicate that participants looked more to animals than to food. Although we should be careful in interpreting this effect, previous studies suggest that such negative FAB may indicate an attentional avoidance behavior (e.g., [39]. Werthmann et al. [39] further suggest that "attentional avoidance of desired stimuli might be a voluntary strategy to resist consumption" (p. 567). In view of this suggestion, it is plausible that the cognitive reappraisal procedure may have elicited such voluntary strategy, causing participants in the CR group to deliberately divert attention from the high-calorie food stimuli, after reminding themselves the negative consequences of consuming such food. Future research that will investigate this hypothesis directly may shed more light on the behavioral changes following CR strategy.

The second hypothesis was that disordered eating would moderate the relationship between CR and FAB. Although such general effect was not found, when we focused on the bulimia scale of the disturb eating questionnaire interesting findings were revealed. The bulimia sub-subscale of the EAT-26 is associated with over-eating [10], and was found to moderate the relationship between CR and FAB. Participants high on bulimia were more susceptible to the intervention, as evidence by the larger pre/post intervention difference they exhibited compared to participants in the CN group. This finding suggests that for people with high levels of overeating, CR may help reduce FAB and, in turn, reduce food intake and weight gain. Danner et al. [7] found that people with disordered eating behaviors based on poor control ability, such as bulimia, made less use of cognitive strategies when compared to others. The use of CR involves restraint and control behaviors [2, 21, 26, 27]. The findings of the present study indicate that when people high on the bulimia sub-scale are instructed to use this strategy, it may increase their strategic use of restraint and control mechanisms.

This study aimed to demonstrate the use of VDP as a tool for assessing FAB and the factors that may moderate it. However further research is required for establishing our innovative findings. One important limitation of our study is that although we tried to recruit participants with a broad weight and BMI range, in practice, we found it difficult to reach many pathologically obese participants. Most of the participants in our study were at on the center of the BMI range (58%), and although we succeeded in recruiting some participants with high BMI values (i.e., overweight and obesity) it was only a small part of our sample (13%). This may explain why we did not get any evidence that the effect of the CR intervention was moderated by BMI. Future research on this topic should include more diverse populations in terms of BMI, especially overweight and obese individuals including more pronounced pathologies, such as eating disorders and obesity.

Another limitation of this study concerns the nature of the CR intervention. We instructed participants to produce five sentences that would make them less appreciative of the food pictures they were viewing. Participants created their own sentences, and we did not supervise these sentences. The advantage of this procedures is that it lets participants choose what they think would be most effective for them. The limitation, in terms of the research is that we have no way of knowing if they would have real value for them in everyday situations. Future studies should examine different sentences to try and underly what kind of sentences are most effective for CR.

Despite these limitations, the current study is innovating in demonstrating that cognitive strategies modulate food related behaviors and in particular that they may reduce attentional bias toward high-calorie foods, and potentially even lead to attentional avoidance from such food. Future studies should extend this investigation to examine the effect of such CR-elicited attentional avoidance on the actual eating behavior, and test to what extent they reduce the consumption of fattening foods. In addition, future studies should examine the effect of time on the effectivity of CR interventions. Here we focused on the immediate and short-term impact of CR on FAB; future research could extend the time frame of the pre/post testing to examine the impact of the CR intervention in the long-term.

In conclusion, the results of the present study have important theoretical implications highlighting the attentional mechanisms underlying food intake, and high calorie foods in particular.

## Conclusions

This study also has practical and clinical implications because using CR to change FAB levels may serve as an intervention to prevent unhealthy eating. The ability to use a relatively simple, easy-to-implement strategy with no financial cost is extremely important in an era in which individuals are constantly solicited to consume high calories food by a variety of stimuli and temptations for unhealthy food consumption. In addition, understanding which individuals respond better to CR can be another step in tailoring a customized plan, and may greatly contribute to coping with reducing the individual's FAB in order to help him maintain his weight.

## Data Availability

All data and materials are available upon request.

## Appendix

See Table 4.

**Table 4:** Means, SDs, and significant t-tests for comparison of lists

t(df)	List A Mean (S.D.)	List B Mean (S.D.)	t(df)
*Words*			
Appealing for eating: animals	1.49 (.80)	1.66 (1.19)	− 2.1 (59)*
Appealing for eating: food	5.64 (.94)	5.70 (.96)	− 1.79 (40)
*Pictures*			
Clear: animals	6.75 (.84)	6.9 (.24)	− 1.27 (56)
Clear: food	6.47 (.78)	6.57 (.83)	− 1.08 (58)
Attract attention: animals	5.28 (1.21)	5.56 (1.25)	− 2.79 (56)*
Attract attention: food	4.86 (1.36)	4.8 (1.28)	.56 (58)
Appealing for eating: animals	1.37 (.77)	1.44 (.83)	− 2.34 (56)*
Appealing for eating: food	5.33 (1.00)	5.35 (.99)	-.17 (58)

**p*<0.05

## Abbreviations

CR: Cognitive reappraisal
FAB: Food attentional bias
VDP: Visual Dot Probe
CN: Control
EAT-26: Eating Attitudes Test Questionnaire
ms.: Milliseconds
RT: Reaction time
AB: Attentional Bias
fMRI: Functional Magnetic Resonance Imaging
BMI: Body mass index

## References

[1] Alcaro A, Huber R, Panksepp J (2007). Behavioral functions of the mesolimbic dopaminergic system: an affective neuroethological perspective. Brain Res Rev.

[2] Appelhans BM (2009). Neurobehavioral inhibition of reward-driven feeding: implications for dieting and obesity. Obesity.

[3] Berridge KC (1996). Food reward: brain substates of wanting and linking. Neurosci Biobehav Rev.

[4] Blechert J, Meule A, Busch NA, Ohla K (2014). Food-pics: an image database for experimental research on eating and appetite. Front Psychol.

[5] Bozarth MA (1994) Pleasure systems in the brain. Pleasure: Polit Reality 5–14‏‏

[6] Broussard JL, Van Cauter E (2016). Disturbances of sleep and circadian rhythms: novel risk factors for obesity. Curr Opin Endocrinol Diabetes Obes.

[7] Danner UN, Evers C, Stok FM, Elburg AA, Ridder DT (2012). A double burden: emotional eating and lack of cognitive reappraisal in eating disordered women. Eur Eat Disord Rev.

[8] Dietrich A (2017) Food craving regulation in the brain: the role of weight status and associated personality aspects (Doctoral dissertation, Max Planck Institute for Human Cognitive and Brain Sciences Leipzig)

[9] Ehrman RN, Robbins SJ, Bromwell MA, Lankford ME, Monterosso JR, O'Brien CP (2002). Comparing attentional bias to smoking cues in current smokers, former smokers, and non-smokers using a dot-probe task. Drug Alcohol Depend.

[10] Garner DM, Olmsted MP, Bohr Y, Garfinkel PE (1982). The eating attitudes test: Psychometric features and clinical correlates. Psychol Med.

[11] Giuliani NR, Calcott RD, Berkman ET (2013). Piece of cake. cognitive reappraisal of food craving. Appetite.

[12] Graham R, Hoover A, Ceballos NA, Komogortsev O (2011). Body mass index moderates gaze orienting biases and pupil diameter to high and low calorie food images. Appetite.

[13] Hendrikse J, Cachia R, Kothe E, McPhie S, Skouteris H, Hayden M (2015). Attentional biases for food cues in overweight and individuals with obesity: A systematic review of the literature. Obes Rev.

[14] Kelley AE, Berridge KC (2002). The neuroscience of natural rewards: relevance to addictive drugs. J Neurosci.

[15] Kemps E, Tiggemann M, Hollitt S (2014). Biased attentional processing of food cues and modification in obese individuals. Health Psychol.

[16] Kober H, Mende-Siedlecki P, Kross EF, Weber J, Mischel W, Hart CL (2010). Prefrontal-striatal pathway underlies cognitive regulation of craving. Proc Natl Acad Sci USA.

[17] Koslowsky M, Scheinberg Z, Bleich A, Mark M, Apter A, Danon Y, Solomon Z (1992). The factor structure and criterion validity of the short form of the Eating Attitudes Test. J Pers Assess.

[18] Littleton HL, Ollendick T (2003). Negative body image and disordered eating behavior in children and adolescents: What places youth at risk and how can these problems be prevented?. Clin Child Fam Psychol Rev.

[19] MacLeod C, Matthews A (2012). Cognitive bias modification approaches to anxiety. Annu Rev Clin Psychol.

[20] May J, Andrade J, Panabokke N, Kavanagh D (2004). Images of desire: cognitive models of craving. Memory.

[21] McKinley NM, Hyde JS (1996). The objectified body consciousness scale: development and validation. Psychol Women Q.

[22] McLean SA, Paxton SJ, Wertheim EH (2010). Factors associated with body dissatisfaction and disordered eating in women in midlife. Int J Eat Disord.

[23] McLean SA, Paxton SJ, Wertheim EH (2011). A body image and disordered eating intervention for women in midlife: a randomized controlled trial. J Consult Clin Psychol.

[24] Mei Z, Grummer-Strawn LM, Pietrobelli A, Goulding A, Goran MI, Dietz WH (2002). Validity of body mass index compared with other body-composition screening indexes for the assessment of body fatness in children and adolescents. Am J Clin Nutr.

[25] Nabavi SF, Russo GL, Daglia M, Nabavi SM (2015). Role of quercetin as an alternative for obesity treatment: you are what you eat!. Food Chem.

[26] Nederkoorn C, Houben K, Hofmann W, Roefs A, Jansen A (2010). Control yourself or just eat what you like? Weight gain over a year is predicted by an interactive effect of response inhibition and implicit preference for snack foods. Health Psychol.

[27] Nederkoorn C, Smulders FT, Havermans RC, Roefs A, Jansen A (2006). Impulsivity in obese women. Appetite.

[28] Nijs IM, Franken IH, Muris P (2010). Food-related Stroop interference in obese and normal-weight individuals: behavioral and electrophysiological indices. Eat Behav.

[29] Nijs IM, Muris P, Euser AS, Franken IH (2010). Differences in attention to food and food intake between overweight/obese and normal-weight females under conditions of hunger and satiety. Appetite.

[30] Ogden CL, Yanovski SZ, Carroll MD, Flegal KM (2007). The epidemiology of obesity. Gastroenterology.

[31] Pinto RM, Steinmetz LS, MG, B J, FCS M A, Curado MP, da Cruz AD (2019). The role of genetics in the pathophysiology of obesity: a systematic review. Obesity Res-Open Jl.

[32] Polivy J, Herman CP, Coelho JS (2008). Caloric restriction in the presence of attractive food cues: external cues, eating, and weight. Physiol Behav.

[33] Rey-Lopez JP, Rezende LF, Pastor-Valero M, Tess BH (2014). The prevalence of metabolically healthy obesity: a systematic review and critical evaluation of the definitions used. Obes Rev.

[34] Siep N, Roefs A, Roebroeck A, Havermans R, Bonte M, Jansen A (2012). Fighting food temptations: the modulating effects of short-term cognitive reappraisal, suppression and up-regulation on mesocorticolimbic activity related to appetitive motivation. Neuroimage.

[35] Stice E, Yokum S, Burger K, Rohde P, Shaw H, Gau JM (2015). A pilot randomized trial of a cognitive reappraisal obesity prevention program. Physiol Behav.

[36] Striegel-Moore RH, Silberstein LR, Frensch P, Rodin J (1989). A prospective study of disordered eating among college students. Int J Eat Disord.

[37] Townshend J, Duka T (2001). Attentional bias associated with alcohol cues: differences between heavy and occasional social drinkers. Psychopharmacology.

[38] Wadden TA, Stunkard AJ (eds) (2002) Handbook of obesity treatment. Guilford Press.‏‏

[39] Werthmann J, Roefs A, Nederkoorn C, Mogg K, Bradley BP, Jansen A (2011). Can (not) take my eyes off it: attention bias for food in overweight participants. Health Psychol.

[40] Werrij MQ, Jansen A, Mulkens S, Elgersma HJ, Ament AJ, Hospers HJ (2009). Adding cognitive therapy to dietetic treatment is associated with less relapse in obesity. J Psychosom Res.

[41] World Health Organization (2015). World health statistics 2015. World Health Organization

[42] Wright SM, Aronne LJ (2012). Causes of obesity. Abdomin Radiol.

[43] Yokum S, Stice E (2013). Cognitive regulation of food craving: Effects of three cognitive reappraisal strategies on neural response to palatable foods. Int J Obes.

[44] Zohar AH, Giladi L, Givati T (2007). Holocaust exposure and disordered eating: a study of multi-generational transmission. Eur Eat Disorders Rev: Profess J Eat Disorders Assoc.

